# Non-propagative human parainfluenza virus type 2 nasal vaccine robustly protects the upper and lower airways against SARS-CoV-2

**DOI:** 10.1016/j.isci.2021.103379

**Published:** 2021-11-17

**Authors:** Junpei Ohtsuka, Masaki Imai, Masayuki Fukumura, Mitsuyo Maeda, Asami Eguchi, Ryoichi Ono, Tadashi Maemura, Mutsumi Ito, Seiya Yamayoshi, Yosky Kataoka, Yoshihiro Kawaoka, Tetsuya Nosaka

**Affiliations:** 1 Department of Microbiology and Molecular Genetics, Mie University Graduate School of Medicine, Tsu 514-8507, Japan; 2 Research Center for Development of Recombinant VLP Vaccines, Research Institutes of Excellence, Mie University, Tsu 514-8507, Japan; 3 BioComo Inc., Komono, Mie 510-1233, Japan; 4 Division of Virology, Department of Microbiology and Immunology, Institute of Medical Science, University of Tokyo, Tokyo 108-8639, Japan; 5 Multi-Modal Microstructure Analysis Unit, RIKEN-JEOL Collaboration Center, Kobe 650-0047, Japan; 6 Laboratory for Cellular Function Imaging, RIKEN Center for Biosystems Dynamics Research, Kobe 650-0047, Japan; 7 Influenza Research Institute, Department of Pathobiological Sciences, School of Veterinary Medicine, University of Wisconsin-Madison, Madison, WI 53711, USA; 8 Department of Special Pathogens, International Research Center for Infectious Diseases, Institute of Medical Science, University of Tokyo, Tokyo 108-8639, Japan

**Keywords:** Infection control in health technology, Virology

## Abstract

We developed an intranasal vaccine against severe acute respiratory syndrome coronavirus 2 (SARS-CoV-2) using the replication-incompetent human parainfluenza virus type 2 (hPIV2) vector BC-PIV, which can deliver ectopic gene as stable RNA and ectopic protein on the envelope. BC-PIV expressing the full-length prefusion-stabilized *spike* gene (K986P/V987P) of SARS-CoV-2, S-2PM, possessed a corona-like viral envelope. Intranasal vaccination of mice with BC-PIV/S-2PM induced high levels of neutralizing immunoglobulin G (IgG) and mucosal IgA antibodies against the spike protein. Although BC-PIV showed hemagglutinating activity, BC-PIV/S-2PM lacked such activity, in accordance with the presence of the massive spike protein on the viral surface. Furthermore, single-dose intranasal vaccination of hamsters with BC-PIV/S-2PM completely protected the lungs from SARS-CoV-2 at 11-week post-immunization, and boost vaccination two weeks before the challenge conferred virtually complete protection of the nasal turbinates against SARS-CoV-2. Thus, this chimeric hPIV2/spike intranasal vaccine is a promising vaccine candidate for SARS-CoV-2 to curtail virus transmission.

## Highlights

• Non-replicating viral vector against spike induces mucosal immunity to block infection
• The viral vector carries spike protein on its envelope with corona-like structure
• One-shot nasal vaccination of hamsters completely protects lungs against severe acute respiratory syndrome coronavirus 2 (SARS-CoV-2)
• Two-shot nasal vaccination of hamsters nearly completely protects the upper airway

## Graphical abstract

**Figure:**
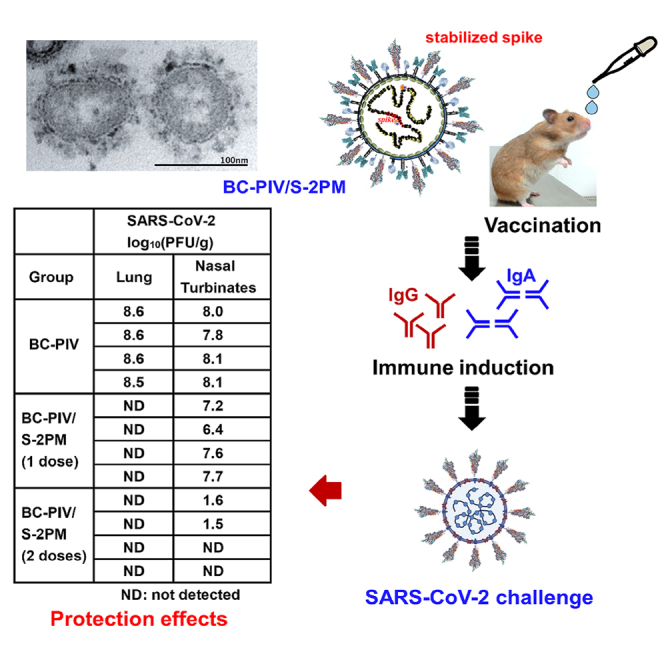

## Introduction

The novel coronavirus severe acute respiratory syndrome coronavirus 2 (SARS-CoV-2) has become a serious threat to people around the world by causing coronavirus disease 2019 (COVID-19). SARS-CoV-2 is one of the most burdensome viruses which have ever become pandemic. Although SARS-CoV and Middle East respiratory syndrome coronavirus (MERS-CoV) do not proliferate in the upper respiratory tract, SARS-CoV-2 attacks both the upper and lower respiratory tracts and sheds viral particles from the throat before symptoms start, spreading easily from person to person. In addition, once SARS-CoV-2 reaches the lungs, it causes severe pneumonia in combination with cytokine release syndrome and/or microvascular disease (Cyranoski, 2020; Zhou et al., 2020; Zhu et al., 2020). Therefore, a nasal vaccine is an ideal method of protecting against SARS-CoV-2 infection at the point of entry, the upper respiratory tract, by inducing mucosal-neutralizing IgA antibodies. Also, to avoid the development of vaccine-associated enhanced respiratory disease (ERD) and antibody-dependent enhancement (ADE) (Graham, 2020), a steric structure-based vaccine design to induce efficiently neutralizing antibodies is mandatory, although such events are not common in SARS-CoV-2 infection. As of September 2021, more than 4 million people had died of COVID-19, and more than 220 million people were infected with SARS-CoV-2. To overcome COVID-19, a safe, efficacious, cost-effective, and easy-to-handle vaccine is crucial.

We recently developed a versatile platform technology to rapidly generate recombinant vaccines following the emergence of a life-threatening new pathogen (Ohtsuka et al., 2019). This technology enabled us to deliver genes and membrane proteins efficiently with a replication-incompetent respiratory viral vector called BC-PIV which is derived from human parainfluenza virus type 2 (hPIV2). hPIV2 is a respiratory virus with little pathogenicity to healthy adults. It is a negative-stranded non-segmented paramyxovirus that causes no antigenic shift. As it is a cytoplasmic virus, it induces no structural alterations of the host genome after infection. It causes recurrent infection throughout a human's lifetime owing to incomplete induction of neutralizing antibodies against hPIV2, or no long-lasting immunity (Vainionpaa and Hyypia, 1994; Henrickson, 2003), possibly enabling multiple administrations of this vector, if required. Of note, the expression of the transgene from BC-PIV is about 100 times higher than that from a conventional retroviral vector in human dendritic cells (Hara et al., 2013). The RNA genome of paramyxovirus is surrounded by a nucleocapsid protein (NP), being free from nucleases, and each NP subunit is associated with precisely six nucleotides of the genome (Kolakofsky et al., 1998). BC-PIV notably has the ability to display basically any type of a membrane-bound gene product on the viral envelope, either as an authentic form or a chimeric form with the transmembrane and cytoplasmic regions of hPIV2 F or HN in either orientation, maintaining the native steric structure of the protein in a sufficient quantity (Ohtsuka et al., 2019). Replication-incompetency was achieved by deleting the vital *F* gene from the hPIV2 genome, and a stable cell line named Vero/BC-F expressing hPIV2 F was established to amplify BC-PIV vectors without detectable mutations during production (Ohtsuka et al., 2014). These properties make BC-PIV an ideal vaccine vector that can be administered intra-muscularly, sub-dermally, or intra-nasally. We have already prepared and stored the master cell bank of Vero/BC-F under GMP control with a serum-free culture system for efficient production (>5×10^8^ TCID_50_/mL) of recombinant BC-PIV.

In the present study, the spike (S) protein of SARS-CoV-2 was used as a vaccine antigen. The S protein is known to be cleaved into S1 and S2 regions by cellular proteases. S1 binds the host cell receptor angiotensin-converting enzyme (ACE) 2 (Hoffmann et al., 2020) and S2 mediates the viral-cell membrane fusion. Binding of the S1 subunit to its receptor destabilizes the wild-type prefusion trimer, resulting in the release of the S1 subunit and a steric structural change in the postfusion conformation of S2 (Walls, et al., 2017; Wrapp et al., 2020). However, S-2P mutations (K986P/V987P) were reported to block the conversion of the S2 protein from a prefusion state to a postfusion state (Pallesen et al., 2017; Kirchdoerfer et al., 2018), disabling the S-mediated proliferation of the recombinant virus. Thus, S-2P mutations retain binding of S1 and prefusion S2 on the BC-PIV virion, while allowing the vector to infect the target cell through the F and HN proteins of hPIV2. Theoretically, the S1 subunit alone with or without membrane anchoring, or a naturally processed complex, S1 on the prefusion S2 as a membrane anchoring protein, would have been expected to be good candidates as antigens for a vaccine against SARS-CoV-2.

Here we created four kinds of recombinant intranasal vaccines using BC-PIV. Among the four constructs, full-length S with prefusion-stabilized 2P mutations, in which the transmembrane and cytoplasmic regions of S2 were replaced by those of F of cognate virus hPIV2, was most immunogenic in mice experiments, and showed dramatic effects to protect upper and lower respiratory tracts of hamsters in SARS-CoV-2 challenge experiments. This BC-PIV vaccine will, therefore, help overcome COVID-19 in the future by herd immunity through preventing infection.

## Results

### Syncytia formation by wild-type spike protein of severe acute respiratory syndrome coronavirus 2 but not by prefusion-stabilized 2P mutant

**Figure 1:**
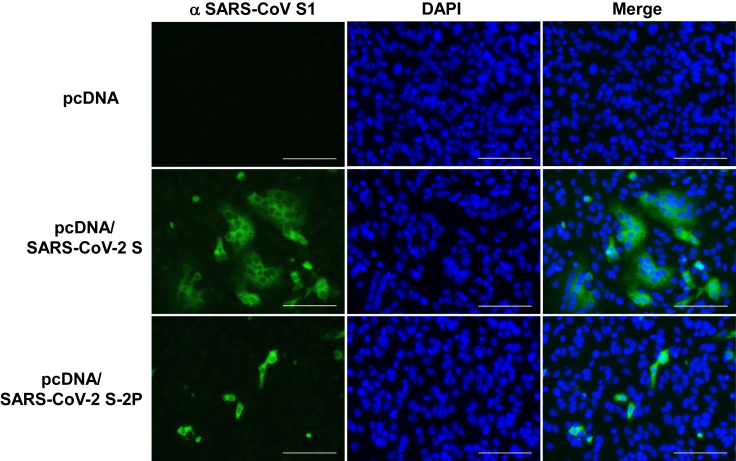
Wild-type S-mediated syncytia formation, but not by prefusion-stabilized S of SARS-CoV-2 Vero cells were transfected with pcDNA expressing the SARS-CoV-2 wild-type S or its 2P mutant, and S1 protein was immunohistochemically stained with Alexa 568 48 h after the transfection. Nuclei were visualized with DAPI. Scale bars, 100 μm.

First, we transfected the full-length wild-type *S* gene into Vero cells to examine the induction of syncytium formation. Similar to the previous finding with the infection of recombinant parainfluenza virus 5 (PIV5) expressing MERS-CoV S to Vero cells (Li et al., 2020), massive syncytia were formed by SARS-CoV-2 S expression. In contrast, the expression of SARS-CoV-2 S-2P mutant did not generate syncytia in Vero cells. Immunohistochemical analyses with an anti-SARS-CoV S1 antibody cross-reactive to SARS-CoV-2 S1 confirmed the wild-type S protein-mediated syncytia formation, but the S-2P mutant lacked the ability to induce syncytia (Figure 1) as expected. Lack of cell fusion indicates lack of infectivity by S-mediated virus-cell fusion. These findings suggest that the prefusion-stabilized double mutations in an S2 region of the full-length S abolish the S-mediated infectivity, without any visible cytopathic effects, including syncytia formation. Inability of infection through S protein contributes to replication-incompetency of BC-PIV/S together with limitation to single-round infection of BC-PIV.

### Construction of recombinant BC-PIV vaccines

**Figure 2:**
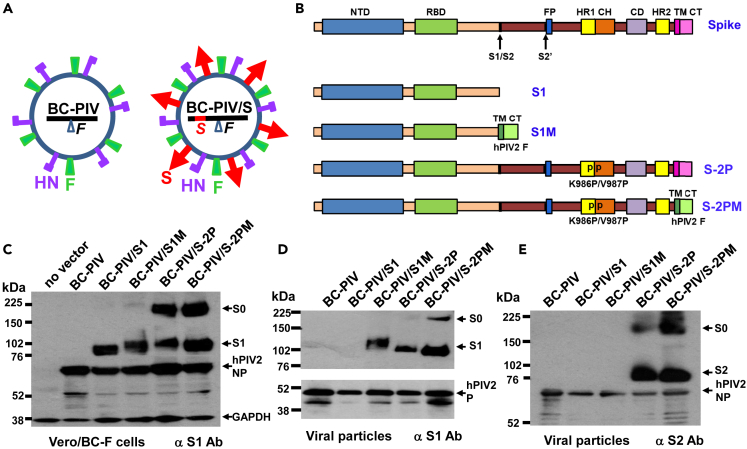
Four kinds of vaccine candidates against SARS-CoV-2 (A) Schematic illustration of a BC-PIV-based chimeric vaccine. The *Spike* (*S*) gene of SARS-CoV-2 is inserted at cloning site 1 of the BC-PIV (Ohtsuka et al., 2019) to enable the high expression of *S* relative to the downstream genes. S protein (S1M, S-2P, and S-2PM; see the panel (B) is incorporated in the viral particle as an envelope protein, resulting in the chimeric virus. (B) A diagram of the transgene cassettes of four vaccine candidates. S, full-length wild-type S of SARS-CoV-2 for reference; NTD, N-terminal domain; RBD, receptor-binding domain; S1/S2, S1/S2 protease cleavage site, S2′, S2′ protease cleavage site; FP, fusion peptide; HR1, heptad repeat 1; CH, central helix; CD, connector domain; HR2, heptad repeat 2; TM, transmembrane domain; CT, cytoplasmic tail; PP, two proline mutations at amino acid positions K986 and V987 in the S2 region to stabilize S in the prefusion conformation; S1, entire region of S1; S1M, S1 fused with the transmembrane and cytoplasmic tail regions of hPIV2 F protein; S-2P, prefusion-stabilized full-length spike protein; S-2PM, ectodomain of S-2P fused with TM and CT regions of hPIV2F. (C) A Western blot analysis of the packaging cells infected with each vaccine vector and probed with anti-SARS-CoV S1, anti-NP of hPIV2, and anti-glyceraldehyde 3-phosphate dehydrogenase (GAPDH) antibodies. S0, uncleaved S protein. (D) A Western blot analysis of the viral particles of each vaccine vector probed with the anti-SARS-CoV S1 and anti-P of hPIV2 antibodies. (E) A Western blot analysis of the viral particles of each vaccine vector probed with the anti-SARS-CoV S2 and anti-NP of hPIV2 antibodies.

BC-PIV is a replication-incompetent platform vector derived from hPIV2. As BC-PIV lacks the *F* gene of hPIV2, it proliferates only in the presence of F protein supplied *in trans*, such as in a packaging cell line Vero/BC-F that stably expresses F protein (Ohtsuka et al., 2014). BC-PIV is able to carry ectopic gene products on its envelope as membrane-bound proteins in the form of a pseudotype virus (Figure 2A) (Ohtsuka et al., 2019).

As there was no syncytium induction by the S-2P mutant, four candidates for a SARS-CoV-2 vaccine were generated using the BC-PIV vector. The inserts and names of the resultant plasmids were as follows: entire S1 (1–681 amino acids) of SARS-CoV-2, BC-PIV/S1; entire S1 (1–681) followed by transmembrane and cytoplasmic tail regions of hPIV2 F (682–740), BC-PIV/S1M; full-length S (1–1273) with double mutations (K986P/V987P), BC-PIV/S-2P; and ectodomain (1–1213) of S-2P fused with transmembrane and cytoplasmic regions of hPIV2 F (1214–1272), BC-PIV/S-2PM (Figure 2B). S1M, S-2P, and S-2PM were supposed to be anchored to the envelope of BC-PIV as a membrane-bound protein in a trimer form. BC-PIV/S1 was considered to produce a secretory S1 protein as a monomer without being loaded on the viral surface.

### Incorporation of the S1 protein onto the BC-PIV virion

The results of the Western blot analyses on the transgene expression from each construct in Vero/BC-F cells after infection were as expected (Figure 2C). In addition, the viral particles recovered from the supernatant of Vero/BC-F cells after infection with each vector were shown to keep the transgene product S1 on the virion, except for BC-PIV/S1, which produces soluble protein S1 (Figure 2D). Secretion of S1 from BC-PIV/S1-infected cells into the medium was demonstrated by analyzing the culture supernatant of Vero cells (Figure S1). The S2 expression by the S-2P and S-2PM constructs was also confirmed on viral particles (Figure 2E). BC-PIV/S-2PM showed better incorporation of S protein in the viral particle than BC-PIV/S-2P, probably owing to the replacement of the transmembrane and cytoplasmic regions of the S protein by those of the F protein of the cognate virus.

**Figure 3:**
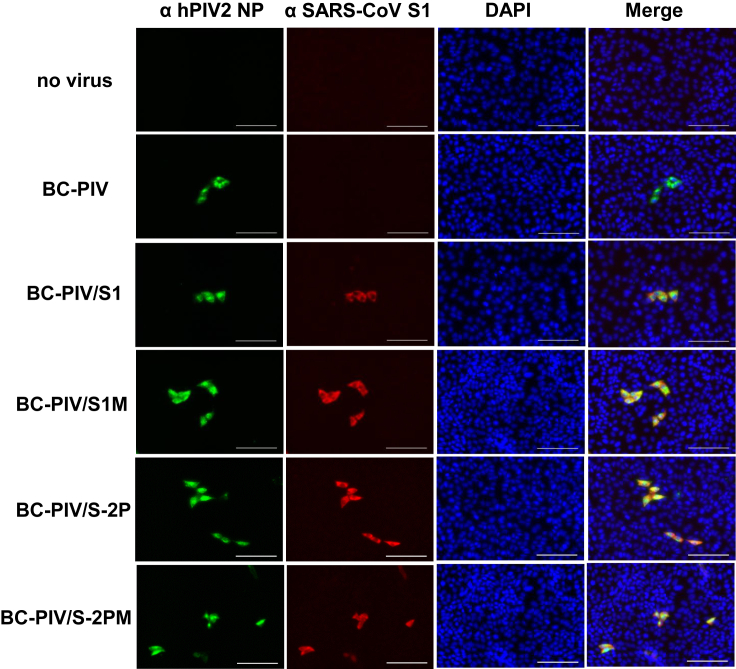
The expression of four kinds of SARS-CoV-2 S in Vero cells 3 days after infection with each BC-PIV vaccine vector at the MOI of 0.3 S1 of SARS-CoV-2, NP of hPIV2, and nuclei were immunohistochemically stained with Alexa 568, Alexa 488, and DAPI, respectively. Scale bars, 100 μm.

Immunohistochemical analyses of the Vero cells after infection with each viral vector using the anti-SARS-CoV S1 antibody are shown in Figure 3. None of the four constructs induced syncytia or the diffuse distribution of the S1 protein among neighboring cells, suggesting in vitro safety of these vaccine constructs owing to the replication incompetency of BC-PIV and the 2P mutations in the two constructs with full-length S.

### Corona-spike formation on the BC-PIV envelope revealed by electron microscopy

**Figure 4:**
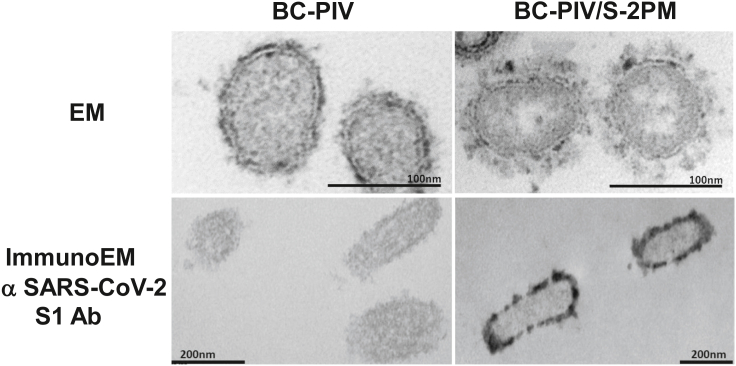
Corona-spike formation on the envelope of the chimeric hPIV2/S vaccine vector Upper panels: conventional electron microscopy of the vector-infected Vero/BC-F packaging cells that were fixed with paraformaldehyde and glutaraldehyde to observe the fine structure of the viral surface. Lower panels: immunoelectron microscopy of the cells of the same preparation as in the upper panels, except that paraformaldehyde with a much lower concentration of glutaraldehyde was used for fixation of the cells in order to retain the antigenicity. The virion of hPIV2 varies in size (average diameter is between 150 and 300 nm) and shape (Vainionpaa and Hyypia, 1994). Scale bars are shown in each panel.

Electron microscopy of the Vero/BC-F cells after the infection of BC-PIV/S-2PM revealed the coronavirus spike-like structure on the surface of the viral particles released from the cells. A similar structure was not found on the cells infected with the parental BC-PIV, although a much smaller spike-like structure derived from hPIV2 F and HN proteins was noted (upper panels of Figure 4). Immunoelectron microscopy corroborated the presence of the SARS-CoV-2 S antigen (S1) on the viral particles which were released from the Vero/BC-F cells infected with BC-PIV/S-2PM, but not on those infected with the parental BC-PIV (lower panels of Figure 4).

### Binding to human angiotensin-converting enzyme 2 by S1 protein of severe acute respiratory syndrome coronavirus 2 expressed on the BC-PIV envelope

**Figure 5:**
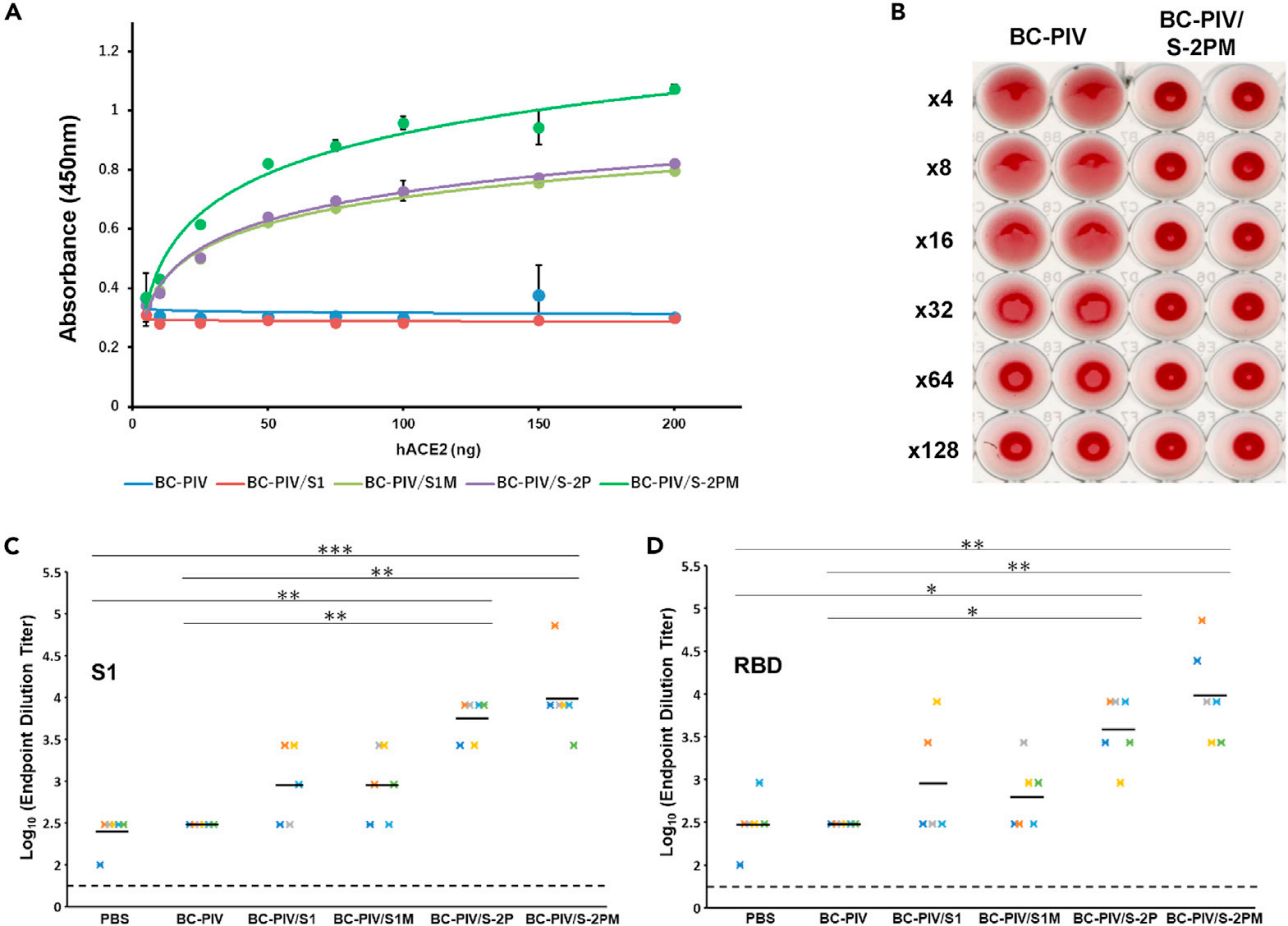
BC-PIV/S-2PM is the best vaccine against SARS-CoV-2 among the four candidates (A) Binding assay between the S1 protein expressed on the viral envelope of each vaccine candidate and recombinant soluble human ACE2. The particles of each vaccine vector were immobilized on a 96-well plate, and the recombinant hACE2 bound to the vector was detected by ELISAs. Averages and standard deviations of triplicate samples are shown. BC-PIV/S-2PM showed the strongest binding to hACE2. Two independent experiments gave the same results, and one representative result is shown. (B) Inhibition of BC-PIV-mediated hemagglutination in the presence of S protein on the envelope of BC-PIV/S-2PM. Inhibition of hemagglutination results in a clear precipitate of erythrocytes, as with the maximally diluted BC-PIV. Three independent experiments gave similar results and one representative result is shown. (C) ELISAs of the sera from the vaccinated mice using the recombinant S1 protein of SARS-CoV-2. Mice were intranasally immunized with one shot of the vector of 2×10^7^ TCID_50_, and blood for an ELISA was drawn on day 33 after vaccination (n = 6 for each construct). The endpoint dilutions of the antibody titer of each mouse in ELISAs are shown. Bars indicate mean values. The Kruskal–Wallis test with the Dunn's post hoc test with Holm–Bonferroni p value adjustment was used. ∗∗p < 0.01, ∗∗∗p < 0.001. (D) ELISAs using the recombinant RBD of S1 and the same mouse sera as in Panel C (n = 6 in each construct). The endpoint dilutions of the antibody titer of each mouse in ELISAs are shown. Bars indicate mean values. The Kruskal–Wallis test with the Dunn's post hoc test with Holm–Bonferroni p value adjustment was used. ∗p < 0.05, ∗∗p < 0.01.

We next investigated the ability of each vaccine candidate to bind to human (h) ACE2 using the viral particles immobilized on the plate in enzyme-linked immunosorbent assays (ELISAs). BC-PIV/S-2PM showed the strongest binding to hACE2 among the four candidates (Figure 5A). BC-PIV/S1 did not show any binding activity to hACE2, as expected, because S1 without S2 is released from the envelope unless transmembrane and cytoplasmic tail regions are provided.

### The spike expression on the envelope abrogates hemagglutination by BC-PIV

To determine the viral titer produced from BC-PIV/S-2PM, a hemagglutination assay was performed. Interestingly, BC-PIV/S-2PM displayed no hemagglutinating activity while BC-PIV showed the activity as in wild-type hPIV2 (Figure 5B). Ectopically expressed massive S protein on the envelope of BC-PIV may have disturbed the interaction of erythrocytes and HN protein of hPIV2. This finding is rather favorable to BC-PIV/S-2PM as a vaccine by precluding possible adverse effects.

### Induction of immunoglobulin G antibodies against severe acute respiratory syndrome coronavirus 2 protein

To examine whether or not BC-PIV-based vaccine vector can elicit humoral immune responses in mice, BALB/c mice were vaccinated via an intranasal route with a single dose of 2×10^7^ median tissue culture infective dose (TCID_50_) of recombinant BC-PIV per mouse. ELISAs using purified S1 protein revealed that high titers of S1-specific immunoglobulin (IgG) antibodies were induced in mouse sera after vaccination with each construct (Figure 5C). ELISAs using the purified receptor-binding domain (RBD) of S1 also gave similar results (Figure 5D). Among the four vaccine candidates, BC-PIV/S-2PM showed the strongest activity for eliciting humoral immunity, consistent with the finding of binding ability to hACE2. Interestingly, BC-PIV/S1 showed stronger antigenic activity than BC-PIV/S1M for inducing antibodies recognizing RBD. We selected BC-PIV/S-2PM as a final vaccine candidate against SARS-CoV-2. Genetic stability of the genome of BC-PIV/S-2PM was examined by reverse transcription-PCR followed by sequencing analyses after 10 passages of the viruses in Vero/BC-F cell culture. The insert sequence was found to have no genetic alterations, and the genomic integrity of the vector was also confirmed as described previously (Ohtsuka et al., 2014) (data not shown).

### Induction of neutralizing antibody and mucosal immunity against severe acute respiratory syndrome coronavirus 2 in intranasally vaccinated mice

**Figure 6:**
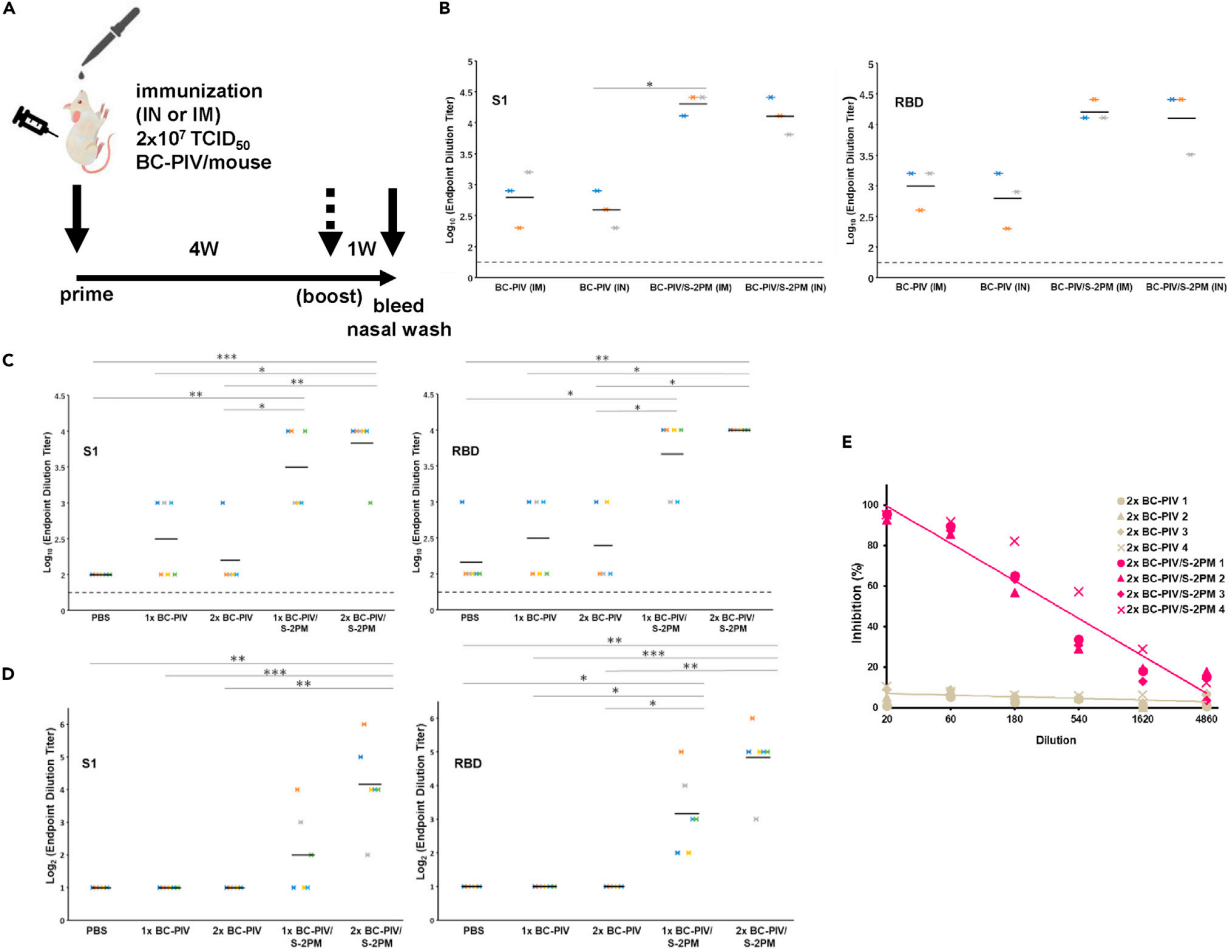
Intranasal immunization of mice with BC-PIV/S-2PM elicited nasal wash IgA antibody against RBD of SARS-CoV-2 S, together with neutralizing IgG antibody against RBD of the S IN, intranasal; IM, intramuscular administration. (A) Timeline and protocol of the vaccination. Four independent experiments gave similar results, and one representative result is shown in panels (B–E). (B) A comparison of the efficacy of the vaccine injection route. One-shot vaccination was performed for serum IgG ELISAs with S1 and RBD, respectively, at day 28 after immunization (n = 3 for each group). IM, intramuscular route; IN, intranasal route. The endpoint dilutions of the antibody titer of each mouse in ELISAs are shown. Bars indicate mean values. The Kruskal–Wallis test with the Dunn's post hoc test with Holm–Bonferroni p value adjustment was used. ∗p < 0.05. (C) Prime and boost intranasal vaccination of mice with BC-PIV/S-2PM elicits a higher serum IgG antibody titer against S1 and RBD, respectively, than single-shot vaccination (n = 5 or 6 for each group). 1x, prime alone; 2x, homologous prime and boost. The endpoint dilutions of the antibody titer of each mouse in ELISAs are shown. Bars indicate mean values. The Kruskal–Wallis test with the Dunn's post hoc test with Holm–Bonferroni p value adjustment was used. ∗p < 0.05, ∗∗p < 0.01, ∗∗∗p < 0.001. (D) Prime and boost intranasal vaccination of mice with BC-PIV/S-2PM elicits higher nasal wash IgA antibody titers against S1 and RBD, respectively, than single-shot vaccination. 1x, prime alone; 2x, homologous prime and boost. PBS (700 μL) was used to flush the nasal mucosa in each vaccinated mouse and then subjected to further dilution for an ELISA (n = 5 or 6 for each group). The endpoint dilutions of the antibody titer of each mouse in ELISAs are shown. Bars indicate mean values. The Kruskal–Wallis test with the Dunn's post hoc test with Holm–Bonferroni p value adjustment was used. ∗p < 0.05, ∗∗p < 0.01, ∗∗∗p < 0.001. (E) The induction of serum neutralizing activity from intranasally vaccinated mice was revealed by the inhibition of the binding between hACE2 and RBD of SARS-CoV-2 S (SARS-CoV-2 surrogate virus neutralization test). Homologous prime and boost immunization (2x) was carried out (n = 4 for each group). The numbers 1–4 represent each individual mouse.

To examine the ability of BC-PIV/S-2PM to elicit the neutralizing antibody against SARS-CoV-2, mice were immunized through a nasal or an intramuscular route on the timeline shown in Figure 6A. The efficiency of IgG antibody induction was nearly equal between intranasal and intramuscular administration at the dose administered (Figure 6B), and no obvious adverse events were observed in vaccinated mice. Hereafter, the intranasal route was selected.

ELISAs of serum IgG and nasal wash IgA were performed to examine the induction of anti-S1 and anti-RBD antibodies after the intranasal vaccination of the mice (Figures 6C and 6D). Nasal wash ELISAs revealed the presence of anti-S1 and anti-RBD IgA antibodies, indicating the induction of nasal mucosal immunity (Figure 6D). The homologous prime and boost protocol were more efficient than a single administration in both IgG and IgA induction for both antigens. This may be owing to the poor transcription of hPIV2 in mice, which are not a natural host (Ito et al., 1989). Next, inhibition assays for the interaction between SARS-CoV-2 RBD and hACE2 by ELISAs as SARS-CoV-2 surrogate neutralization (cPass kit) tests (GenScript) were carried out. This assay was previously demonstrated to be well correlated with both the conventional and pseudovirus-based neutralizing tests against SARS-CoV-2, and the cut-off value of this assay was shown to be 30% inhibition (Tan et al., 2020). Sera from BC-PIV/S-2PM intranasally vaccinated mice showed efficient inhibitory activities for the interaction between SARS-CoV-2 RBD and hACE2, while those from empty vector-vaccinated mice did not (Figure 6E), suggesting the neutralizing activity of the sera against S protein of SARS-CoV-2.

### Modest induction of cellular immunity against severe acute respiratory syndrome coronavirus 2 S protein

SARS-CoV-2 S-specific T cell responses in splenocytes from mice intranasally vaccinated with two shots of BC-PIV/S-2PM were evaluated by measuring intracellular IFN-γ with FACS analyses after 6 h of stimulation with a peptide library spanning the full-length S protein of SARS-CoV-2. Only a modest increase in IFN-γ-producing CD4+ cells was observed in the vaccinated mice compared with the empty vector-treated mice (Figure S2), and IFN-γ-producing CD8+ cells were not significantly increased in the vaccinated mice (data not shown). These results may reflect that mice are not permissive for hPIV2 transcription (Ito et al., 1989), which is essential for inducing cellular immunity.

### Complete protection of the lungs against SARS-CoV-2 challenge in hamsters after single-dose intranasal vaccination with BC-PIV/S-2PM and nearly complete protection of the nasal turbinates after two-shot vaccination

**Figure 7:**
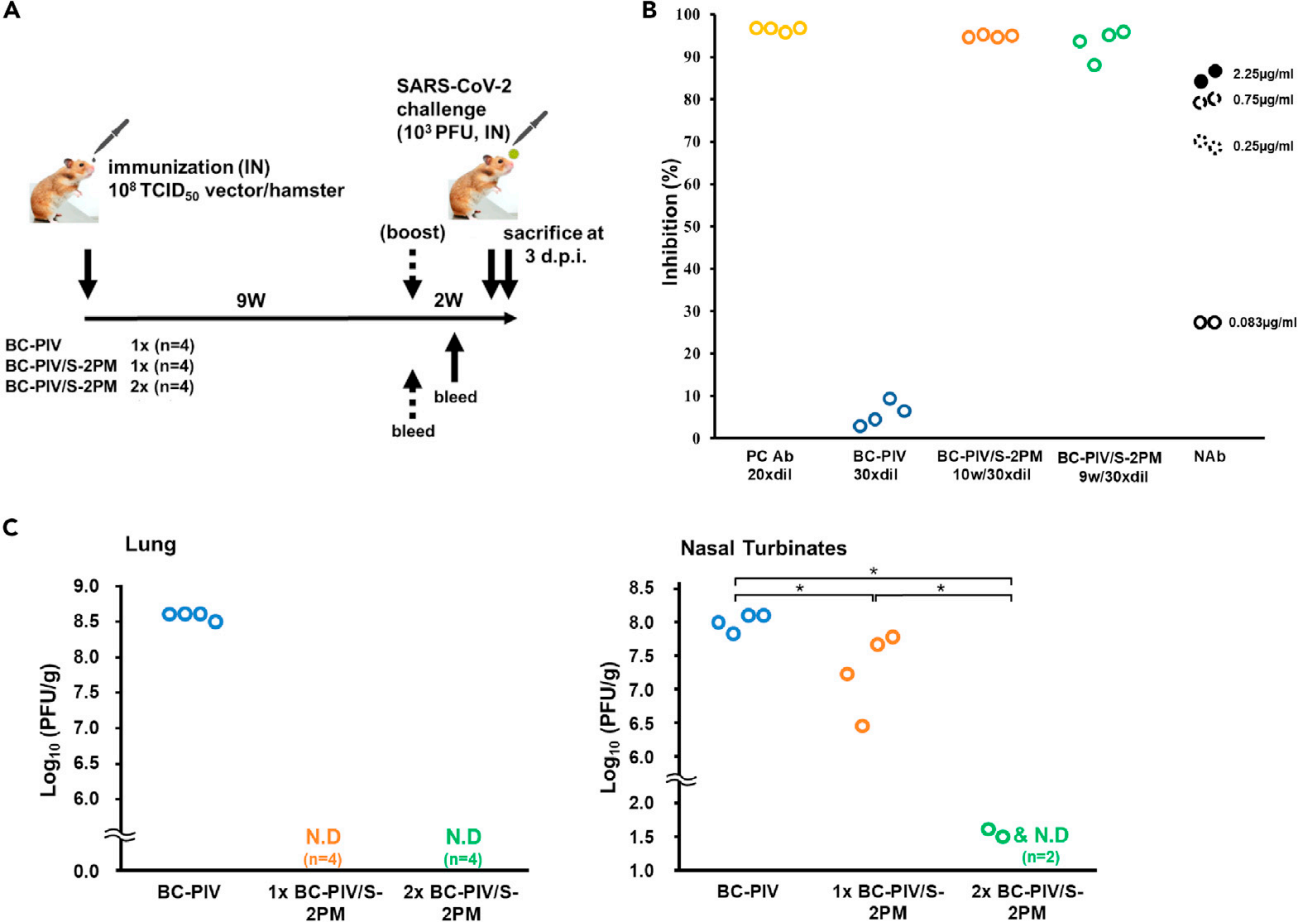
Robust protective effects of BC-PIV/S-2PM vaccine against SARS-CoV-2 infection in hamsters (A) Timeline and protocol of the vaccination and SARS-CoV-2 challenge test. Four hamsters were used for each group. (B) Eminent neutralizing activity of the sera from intranasally vaccinated hamsters after one-shot vaccination was revealed using the SARS-CoV-2 surrogate virus neutralization test. Serum was collected 9 weeks after priming in the prime-boost group and 10 weeks after vaccination in the one-shot group and control group. Hamster sera were analyzed after 15x dilution (final dilution was 30x). PC Ab, positive control serum, serum from a mouse repeatedly immunized with the recombinant RBD protein, which is included in the cPass™ Technology kit (SARS-CoV-2 surrogate virus neutralization test kit). The mouse serum was analyzed after 10x dilution (final dilution was 20x). N Ab, commercially available recombinant human anti-SARS-CoV-2 neutralizing antibody (SAD-S35; ACROBiosystems) as a reference, derived from a patient infected with SARS-CoV-2. The neutralizing antibody was used at the final concentrations indicated. According to the manufacturer, the neutralizing antibody inhibits the interaction between SARS-CoV-2 RBD and hACE2 with an IC_50_ of 1.47 μg/mL when used with the SARS-CoV-2 inhibitor screening kit (ACROBiosystems). (C) A plaque assay at 3-days post-infection of SARS-CoV-2. Infectious viruses in the lungs and nasal turbinates of the hamsters were measured on VeroE6/TMPRSS2 cells (n = 4 hamsters in each group). This plaque assay detects even from one infectious virus. The lower limit of detection (LOD) in this plaque assay depends on the quantity of the tissue used for the analysis. For example, if 1 g of tissue is used, the lower LOD is 0.0 (log_10_(1/1)). N.D., not detected (not plotted in the figure). The Kruskal–Wallis test with the Steel-Dwass post hoc test was used. ∗p < 0.05.

We recently showed that Syrian hamsters are a good model for SARS-CoV-2 infection and pathogenesis experiments (Imai et al., 2020) and are permissive for hPIV2 infection and transgene expression from BC-PIV (hPIV2ΔF) (Ohtsuka et al., 2014). We performed the SARS-CoV-2 challenge test in Syrian hamsters on a timeline shown in Figure 7A. Nine to 10 weeks after the initial immunization without boosting, we confirmed the strong inhibitory activity of the sera of all vaccinated hamsters in the SARS-CoV-2 surrogate virus neutralization test, which is as strong as a positive control serum provided in the cPass kit (Figure 7B). No visible adverse events occurred in vaccinated hamsters. We also evaluated the efficacy of the vaccine by measuring infectious viral burden of SARS-CoV-2 in the lungs and nasal turbinates of the hamsters 3 days after the challenge test. The challenge test disclosed that single-dose intranasal immunization of BC-PIV/S-2PM was sufficient to completely protect the lungs of the hamsters against SARS-CoV-2 at 11 weeks after vaccination with more than a 10^8^-fold reduction in the infectious virus burden compared with those vaccinated with the empty vector, and the nasal turbinates had a significantly reduced viral burden. Although we used 10^8^ TCID_50_ of the vector per hamster for vaccination, we will be able to precisely optimize the best dose of BC-PIV/S-2PM, because the vector never proliferates in vivo (Ohtsuka et al., 2014). Moreover, prime-boost immunization induced nearly complete clearance of the infectious virus in the nasal turbinates with more than a 10^6^-fold reduction in the viral burden compared with the controls, in addition to complete clearance of the virus in the lungs at day 3 after the challenge, which was performed 11-week post-priming (Figure 7C). These findings suggest that strong nasal mucosal immunity in addition to systemic neutralizing antibodies was elicited by two-shot vaccination of BC-PIV/S-2PM, resulting in protection from SARS-CoV-2 infection at the point of entry.

### Effect of a nasal booster vaccination in hamsters 8 months after the priming

**Figure 8:**
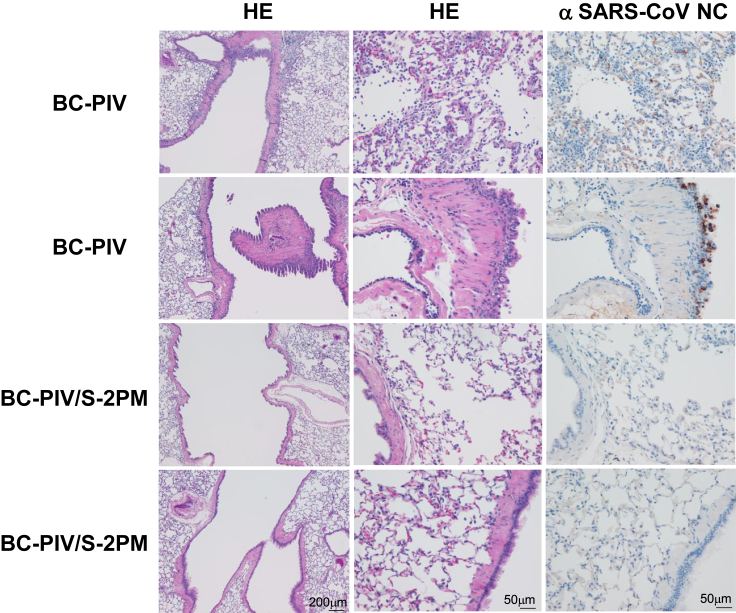
Pathological findings in vaccinated hamsters 3 days after infection with SARS-CoV-2 (Left and middle column panels) Two hamsters after prime (at the age of 5 weeks) and boost (8 months after the priming) vaccination with BC-PIV/S-2PM and those with BC-PIV were challenged with 10^3^ PFU of SARS-CoV-2/head (UT-NCGM02/Human/2020/Tokyo) 3 weeks after the boosting. The lungs of the hamsters were stained with hematoxylin and eosin. (Right column panels) Immunohistochemical staining with an anti-SARS-CoV-1 nucleocapsid protein antibody which cross-reacts with SARS-CoV-2 nucleocapsid protein. Panels on each column are shown with the same magnification. Scale bars are shown in each bottom panel.

To investigate the durability of the antibody responses and the effect of the later booster vaccination on the hamsters that had a single-shot vaccination, two hamsters of the priming alone group were boosted 8 months after the priming, whereby the hamsters were bled on the day of boosting, and the SARS-CoV-2 surrogate neutralization tests using the sera were performed. The serum (40 x dilution) of the one hamster showed 65% inhibition of the binding between the RBD and hACE2, and the serum (40 x dilution) of the other hamster showed 53% inhibition, while the sera (40 x dilution) from the empty vector-administered two hamsters showed 0% inhibition, and the positive control antibody (40 x dilution) showed 92% inhibition. These immunized two hamsters together with the empty vector-administered two hamsters were challenged with SARS-CoV-2 as in the experiments shown in Figure 7, except that 3 weeks after the booster vaccination, and the lungs of the hamsters were histologically analyzed. As shown in Figure 8, histological analyses 3-days post-challenge revealed that macrophages and lymphocytes were infiltrated in the bronchi, bronchioli, and alveolar spaces of the lungs of the BC-PIV-treated hamsters. In addition, SARS-CoV-2 viral antigen was detected in the bronchial/peribronchial and damaged alveolar regions, while prime-boost-vaccinated hamsters showed no damages to the lungs and absence of the viral antigen. Importantly, no infiltration of the inflammatory cells such as neutrophils, eosinophils, and lymphocytes was observed in the alveolar and bronchial regions of the BC-PIV/S-2PM-vaccinated hamsters. Consistent with these findings, the two hamsters with late booster vaccination showed the absence of SARS-CoV-2 in the lung by plaque-forming assay, while the two hamsters treated with BC-PIV showed 8.65 and 8.64 (log_10_(PFU/g)). The sera of the two hamsters 3 weeks after late booster vaccination showed 160 and 40 by the authentic SARS-CoV-2 neutralization assay (Imai et al., 2021) against UT-NCGM02/Human/2020/Tokyo, while the two hamsters treated with BC-PIV showed <10 and <10, respectively. These findings corroborated the protective effects of the nasal vaccine BC-PIV/S-2PM without visible adverse effects such as ADE and ERD. Also, prime vaccination with BC-PIV/S-2PM 8 months prior to the booster vaccination was suggested to give no negative impact upon booster effects of the vaccine, owing to immune reactions against BC-PIV vector itself.

### Neutralizing activity of BC-PIV/S-2PM against severe acute respiratory syndrome coronavirus 2 variants of concern in mice

Finally, it is important to investigate the ability of BC-PIV/S-2PM to neutralize SARS-CoV-2 variants of concern (VoCs). To this end, we used the sera (the same ones used in Figure 6E) from the mice intranasally vaccinated with two shots of BC-PIV/S-2PM or BC-PIV for the neutralization assays against the authentic viruses Wuhan strain (NC002), β strain (B.1.351; MD-HP0154), and δ strain (B.1.617.2; UW-5250). Although the surrogate neutralization assay was extremely sensitive, the authentic virus neutralization assay gave nearly similar results (Table 1) to that shown in Figure 6E except that vaccinated mouse serum #2 exhibited the activity below the detection level of the authentic assay. This unexpectedly low titer of serum #2 may reflect the non-permissive infection of hPIV2 in mice. Interestingly, vaccinated mouse serum #4 neutralized three kinds of viruses with equal efficiency. However, sera #1 and #3 showed stronger activities against the Wuhan strain than those against the other strains. Further analyses using the sera from the vaccinated hamsters will clarify the efficacy of this vaccine against SARS-CoV-2 VoCs.

**Table 1:** Neutralizing activity of BC-PIV/S-2PM against SARS-CoV-2 VoCs in mice

Intranasal vaccine used	Animal ID	Wuhan strain NC002	β strain B.1.351 MD-HP0154	δ strain B.1.617.2 UW-5250
BC-PIV	#1	<20	<20	<20
	#2	<20	<20	<20
	#3	<20	<20	<20
	#4	<20	<20	<20
BC-PIV/S-2PM	#1	40	20	<20
	#2	<20	<20	<20
	#3	80	<20	20
	#4	80	80	80

Mice were intranasally vaccinated with 2 shots of BC-PIV or BC-PIV/S-2PM as shown in Figure 6A. The sere used in Figure 6E were used for authentic SARS-CoV-2 neutralizing assay against SARS-CoV-2 Wuhan strain and variants of concern (VoCs). Virus neutralization titers are shown as the reciprocal of the highest serum dilution that completely prevented the viral cytopathic effects.

## Discussion

The immediate deployment of a protective vaccine against SARS-CoV-2 is an absolute necessity to overcome the COVID-19 pandemic. A rational design for vaccine development is important (Burton and Walker, 2020; Corey et al., 2020; Diamond and Pierson, 2020).

In this study, we employed the prefusion-stabilized S protein (Pallesen et al., 2017; Kirchdoerfer et al., 2018) as a SARS-CoV-2 vaccine antigen for the induction of neutralizing antibodies. A strategy using a stabilized mutant instead of the unstable wild-type protein was employed to target the virus of which surface protein shows the conformational diversity, such as respiratory syncytial virus F protein (McLellan et al., 2013). In case of S protein in SARS-CoV-2, 2P mutations in the S2 region stabilize the S1–S2 complex in prefusion conformation. Furthermore, we replaced the transmembrane and cytoplasmic tail regions of the S protein with those of hPIV2 F protein for better loading of the S protein on the envelope of the BC-PIV (hPIV2ΔF) vector.

It has not escaped our notice that an enveloped viral vector would be extremely useful owing to its ability to carry ectopic proteins on the viral envelope in addition to gene delivery. Vectors such as hPIV2, which has a powerful RNA polymerase, can carry large quantities of protein, based on their efficient expression of the transgene (Hara et al., 2013). Immediate transfer of the protein before translation of the delivered gene would precede the immunological rejection of the vector itself by pre-existing antibody, as in virus-like protein vaccines. On the other hand, we should be cautious to keep replication-incompetency of the enveloped viral vector, because transgene product-mediated viral replication can occur. For example, if we use the wild-type S instead of S-2P, the resultant vector would proliferate even though BC-PIV lacks *F* gene.

Regarding BC-PIV/S-2PM, cross-presentation of the S protein on the vector envelope as well as processed S-derived peptides after translation in antigen-presenting cells would contribute to inducing CTL activity in humans, where hPIV2 genes are to be abundantly transcribed (Hara et al., 2013). In addition, BC-PIV itself has a strong adjuvant activity for inducing human dendritic cell maturation (Hara et al., 2013).

Non-propagative recombinant viral vaccine with an appropriately pseudotyped envelope is considered an ultimate live vaccine with adjuvanticity and safety. It can theoretically elicit innate, cellular, and conventional humoral immunity (Ohtsuka et al., 2019). Furthermore, BC-PIV/S-2PM via intranasal administration can also induce mucosal IgA antibodies to prevent viral infection at the upper respiratory tract, as demonstrated in this study. A recent study on convalescent COVID-19 individuals showed that there was a good correlation between the SARS-CoV-2 pseudovirus-neutralizing activity of IgG and IgA in plasma in a given individual, and clones of IgM-, IgG-, and IgA-producing B cells were found to be derived from common progenitor cells (Wang et al., 2021). It should be noted that prophylactic systemic injection of neutralizing antibody significantly reduces infection in the lungs but not in the nasal turbinates of hamsters intranasally challenged with SARS-CoV-2 (Zhou et al., 2021). Also, SARS-CoV-2 breakthrough infections, associated with large public gatherings, can occur in fully intramuscularly vaccinated persons with similar Ct values of real-time RT-PCR of the specimens to those from unvaccinated persons (Brown et al., 2021).

Intranasal immunization with replication-competent recombinant PIV5 expressing MERS-CoV S protein was previously shown to completely protect mice from lethal MERS-CoV infection, whereas intramuscular immunization with UV-inactivated MERS-CoV with adjuvant protected only 25% of the immunized mice. Furthermore, ERD was observed in lungs of the mice immunized with inactivated MERS-CoV, but not in the mice immunized with PIV5/MERS-CoV-S (Li et al., 2020). Consistent with this is that inactivated SARS-CoV-immunized mice also suffered from ERD after a challenge with SARS-CoV (Bolles et al., 2011; Tseng et al., 2012). Similarly, a chimeric, replication-competent vesicular stomatitis virus (VSV) vaccine vector expressing the SARS-CoV-2 S protein was reported to protect against SARS-CoV-2-mediated pathogenesis in mice via intranasal vaccination, although the viral burden of the nasal wash was not significantly reduced (Case et al., 2020). Interestingly, another replication-competent VSV-ΔG-spike vaccine showed a 10^3^-fold reduction of the viral load of SARS-CoV-2 in nasal turbinates via intramuscular vaccination in challenge experiments in hamsters (Yahalom-Ronen et al., 2020).

In our study, replication-incompetent respiratory viral vector BC-PIV expressing prefusion-stabilized S protein was shown to elicit serum neutralizing IgG and nasal mucosal IgA antibodies against SARS-CoV-2 S after intranasal vaccination of mice without any obvious adverse effects. In fact, a single-dose intranasal vaccination with BC-PIV/S-2PM protected hamsters against SARS-CoV-2 challenge with complete clearance of the virus from the lower respiratory tract at 11 weeks after the immunization (more than a 10^8^-fold reduction in the infectious virus). Furthermore, two-shot immunization of hamsters with BC-PIV/S-2PM induced a 10^6^- to 10^8^-fold reduction in the infectious virus in the upper respiratory tract compared with those treated with the empty vector. As mucosal immunity is a prerequisite as the first-line barrier to SARS-CoV-2 entry before viral spread to the lung, nearly complete protection against the infectious virus in the upper airway after two-shot intranasal vaccination is important to consider the strategy for achieving herd immunity against SARS-CoV-2. In addition, effective protection via two-shot vaccination, including late boosting, suggests the usefulness of multiple administration of BC-PIV vaccine, albeit further experiments will be required to prove this possibility.

Of note, single-dose intranasal administration of adenovirus-vectored vaccine was recently reported to protect the upper and lower respiratory tracts against SARS-CoV-2 in mice (Hassan et al., 2020; Wu et al., 2020) and to protect the upper respiratory tract in ferrets (Wu et al., 2020). In these studies, infectious virus in the lungs or nasal washes of the vaccinated animals was reduced by about 10^3^-fold compared with the controls, and sterilizing immunity against SARS-CoV-2 was suspected to have been achieved (Hassan et al., 2020). However, it should be noted that the neutralizing antibody titer against SARS-CoV-2 was reduced in the early convalescent phase in humans (Long et al., 2020), suggesting the possible requirement of multiple vaccinations. The possibility of antibody generation against the adenoviral vector will need to be addressed if repeated administration of the vector is required to maintain immunity. Irrespective of the types of the vaccine, intranasal vaccination is a promising powerful strategy for inducing sterilizing immunity against SARS-CoV-2. The results of human trials will be carefully evaluated.

At present, intramuscular injection of mRNA vaccines against SARS-CoV-2 S is being urgently used in order to overcome the COVID-19 pandemic. A phase 1 clinical study with the mRNA-1273 vaccine encoding S-2P (K986P/V987P) prefusion-stabilized S protein, made by Moderna, demonstrated promising results with regard to the induction of serum-neutralizing antibody in all participants, albeit with relatively frequent adverse events (Jackson et al., 2020). Furthermore, a phase 3 trial showed striking efficacy (94.1%) of this vaccine (Baden et al., 2021). Similar encouraging results of a phase 1/2/3 trial with the BNT162b2 mRNA vaccine encoding S-2P made by BioNTech and Pfizer were also demonstrated (Walsh et al., 2020; Polack et al., 2020). It is interesting that BC-PIV/S-2PM demonstrated more efficacy than mRNA vaccines showing excellent protective effects against SARS-CoV-2 in clinical use, with regard to the extent of reduction of the viral burden in the lungs and the nasal turbinates in SARS-CoV-2 challenge experiments in animals (Corbett et al., 2020), albeit a direct comparison is difficult between the results of different animal models and different experimental settings.

Although the BC-PIV/S-2PM nasal vaccine we developed is currently in the early stage of development, it has ideal properties, including being an efficient inducer of mucosal immunity after the second vaccination shot, possibly allowing for multiple administrations to elicit IgA and IgG neutralizing antibodies and cellular immunity against SARS-CoV-2 in a natural host of hPIV2. Evaluations for the next stage, including safety testing, as well as production under GMP control, are definitely required to advance to clinical trials as a second-generation vaccine against SARS-CoV-2.

As for the other paramyxovirus-based vaccines against SARS-CoV-2 S, Newcastle disease virus (NDV) vector expressing S, measles virus (MeV) vector expressing S, and parainfluenza virus 5 (PIV5) vector expressing wild-type S with its cytoplasmic tail replaced with that of PIV5 F protein, were recently reported. Chimeric NDV/S as inactivated vaccine (intramuscular injection) (Sun et al., 2020b) or replication-competent live vaccine (intramuscular injection) (Sun et al., 2020a), NDV/S intranasal live-attenuated vaccine (Park et al., 2021), MeV/S live-attenuated vaccines (intraperitoneal injection; half subcutaneous and half intranasal injection) (Hörner et al., 2020; Lu et al., 2021), and replication-competent PIV5/S intranasal mucosal vaccine (An et al., 2021) were shown to be efficacious. PIV5/S mucosal vaccine was reported to prevent viral infection and contact transmission in ferrets. BC-PIV/S-2PM is replication-defective and can be administered via an intranasal route with high effects, making it be a strong candidate as a safe vaccine.

Finally, we should be alert for emerging infectious diseases more fatal than COVID-19. mRNA vaccines are definitely useful for the urgent generation of vaccines against newly emerging pathogens or their variants. Likewise, the BC-PIV platform technology makes it possible to generate infection-preventive vaccine seeds within three weeks once the cloned gene of the key antigenic region in the pathogen becomes available, as in COVID-19 vaccine BC-PIV/S-2PM.

### Limitations of the study

Several limitations associated with the present study warrant mention. Although our results using hamsters to prove the efficacy of the vaccine against SARS-CoV-2 are robust, the majority of the immunological data in this study were obtained from mouse experiments because of the broad availability of reagents for mouse studies. However, hPIV2 transcription is too poor in mice to analyze hPIV2-induced cellular immunity, which mainly depends on viral transcription. Therefore, the vaccine effects in mice shown in this study are likely to be mostly derived from the effects of the ectopically expressed S protein on the viral envelope. This indicates that the relatively strong induction of the neutralizing antibodies in the vaccinated mice except mouse #2 in Table 1 predicts promising efficacy of BC-PIV/S-2PM in humans, who are the natural host of hPIV2, although extensive safety tests are required. Another limitation is that this study demonstrated the efficacy of the vaccine only in limited periods and a small number per group in experiments using hamsters. Further studies will be required to monitor the immune responses over time after intranasal vaccination with BC-PIV/S-2PM in order to establish the durability of the effects.

## STAR★Methods

### Key resources table

**Table:** 

REAGENT or RESOURCE	SOURCE	IDENTIFIER
**Antibodies**		

Anti-SARS-CoV Spike Antibody pAb	Sino Biological Inc.	Cat #40150-V08B1
Anti-SARS-CoV-2 Spike antibody pAb	Sino Biological Inc.	Cat #40591-T62
Anti-SARS-CoV Spike antibody (1A9)	GeneTex	Cat #GTX632604
Anti-SARS-CoV nucleocapsid protein polyclonal rabbit antibody	Prospec	Cat # ANT-180
Anti-hPIV2 NP (20A) mAb	Tsurudome et al. (1989)	N/A
Anti-hPIV2 P (211A) mAb	Tsurudome et al. (1989)	N/A
Anti-hPIV2 P/V (315-1) mAb	Nishio et al. (1997)	N/A
Anti-GAPDH, Monoclonal antibody Peroxidase Conjugated	FUJIFILM Wako Pure chemical	Cat #015-25473
Anti-rabbit IgG, HRP-linked Antibody	Cell Signaling Technology	Cat #7074
Anti-mouse IgG, HRP-linked Antibody	Cell Signaling Technology	Cat #7076
Goat Anti-Mouse IgG H&L Alexa Flour488	Abcam	Cat #Ab150113
Goat Anti-Rabbit IgG H&L Alexa Flour568	Abcam	Cat #Ab175471
HRP-Goat anti mouse IgG	BioLegend	Cat #405306
anti-Mouse-IgA HRP	Southern Biotech	Cat #1040-05
Anti-mouse CD28 (37.51)	BioLegend	Cat #102115
PerCP/Cyanine5.5 anti-mouse CD4 Antibody (GK1.5)	BioLegend	Cat #100434
PerCP/Cyanine5.5 anti-mouse CD8a Antibody (53-6.7)	BioLegend	Cat #100734
PE anti-mouse IFN-γ Antibody (XMG1.2)	BioLegend	Cat #505808
Biotinylated anti-rabbit IgG antibody	Vector Laboratory	Cat #BA-1000-1.5

**Bacterial and virus strains**		

BC-PIV	Ohtsuka et al. (2019)	N/A
BC-PIV/S1	This paper	N/A
BC-PIV/S1M	This paper	N/A
BC-PIV/S-2P	This paper	N/A
BC-PIV/S-2PM	This paper	N/A
SARS-CoV-2 (UT-NCGM02)	Imai et al. (2020)	N/A
SARS-CoV-2 Wuhan strain (NC002)	This paper	N/A
SARS-CoV-2 β strain (B.1.351; MD-HP0154)	This paper	N/A
SARS-CoV-2 δ strain (B.1.617.2; UW-5250)	This paper	N/A

**Biological samples**		

Guinea Pig erythrocyte	Japan Bio Serum	Cat #35-0012

**Chemicals, peptides, and recombinant proteins**		

X-treme GENE HP	Roche	Cat #06366244001
DAPI	Nacalai Tesque	Cat #19174-31
Brefeldin A	Biolegend	Cat #420601
SARS-CoV-2 Spike Glycoprotein	GenScript	Cat #RP30020
SARS-CoV-2 Spike S1 Protein	Sino Biological Inc.	Cat #40591-V02H
SARS-CoV-2 Spike Protein RBD	Sino Biological Inc.	Cat #40592-V02H
Human ACE2 Protein	AcroBiosystems	Cat #AC2-H5257
Dako REAL EnVision Detection System	Dako	Cat #K5007

**Critical commercial assays**		

SARS-CoV-2 Surrogate Virus Neutralization Test Kit	GenScript	Cat #L00847
eBioscience™ Intracellular Fixation & Permeabilization Buffer Set	Thermo Fisher Scientific	Cat #88-8824-00

**Experimental models: Cell lines**		

Monkey: Vero	Riken BRC CELL BANK	Cat #RCB0001
Monkey: Vero/BC-F	Ohtsuka et al. (2014)	N/A
Monkey: VeroE6/TMPRSS2	JCRB	Cat #1819

**Experimental models: Organisms/strains**		

Mouse: BALB/c	Japan CLEA	N/A
Hamster: Syrian	Japan SLC	N/A

**Oligonucleotides**		

pcDNA/SARS-CoV-2 S Fw: CAGTGTGGTGGAATTCATGTTTGTGTTCCTGGTGCTG	This paper	N/A
pcDNA/SARS-CoV-2 S Rv : GATATCTGCAGAATTCTCAGGTGTAGTGCAGTTTCAC	This paper	N/A
Vec/SARS-CoV-2 Fw 1: TCTCTATCTGCGGCCGCATATATGTTTGTGTTCCTGGTGCTG	This paper	N/A
Vec/SARS-CoV-2 Fw 2: TCTCTATCTGCGGCCGCAATGTTTGTGTTCCTGGTGCTG	This paper	N/A
Vec/SARS-CoV-2 Rv: AATAGAGATGCGGCCGCCTAACCCGTCCGGGCCTATG	This paper	N/A
SARS-CoV-2 S1/S2 Fw: GAGGGCAAGGTCTGTGGCAAGCCAGAGCATC	This paper	N/A
SARS-CoV-2 S1/S2 Rv: TTGCCACAGACCTTGCCCTCCTTGGGCTGTTG	This paper	N/A
SARS-CoV-2 S2P Fw: ACTGGACCCCCCCGAGGCTGAGGTCCAGATTGAC	This paper	N/A
SARS-CoV-2 S2P Rv: CAGCCTCGGGGGGGTCCAGTCTGCTCAGGATGTC	This paper	N/A
PIV2 F/M Fw: ATAGCATTAATACTATCAGTG	This paper	N/A
SARS-CoV-2 S1/PIV2 F /M Rv: CACTGATAGTATTAATGCTATTGGGCTGTTGGTCTGGGTCTG	This paper	N/A
SARS-CoV-2 S/PIV2 F/M Rv: ACTGATAGTATTAATGCTATTGGCCACTTGATGTATTGTTC	This paper	N/A
PIV2 IG Fw: CTCTCATAATTTAAGAAAAAATC	This paper	N/A
SARS-CoV-2 S1/PIV2 IG Rv: TTTTTCTTAAATTATGAGAGTTATGGGCTGTTGGTCTGGGTC	This paper	N/A
SARS-CoV-2 S/PIV2 IG Rv: TTTTTCTTAAATTATGAGAGTCAGGTGTAGTGCAGTTTCAC	This paper	N/A

**Recombinant DNA**		

pCMV3-2019-nCoV-S1-long	Sino Biological Inc.	Cat #VG40591-UT
pCMV3-2019-nCoV-S2-long	Sino Biological Inc.	Cat #VG40590-UT
pcDNA3.1	Invitrogen	Cat #V79020
pcDNA-SARS-CoV-2 S	This paper	N/A
pcDNA-SARS-CoV-2 S-2P	This paper	N/A
phPIV2ΔF (BC-PIV)	Ohtsuka et al. (2014)	N/A
phPIV2ΔF-SARS-CoV-2 S1 (BC-PIV/S1)	This paper	N/A
phPIV2ΔF-SARS-CoV-2 S1M (BC-PIV/S1M)	This paper	N/A
phPIV2ΔF-SARS-CoV-2 S-2P (BC-PIV/S-2P)	This paper	N/A
phPIV2ΔF-SARS-CoV-2 S-2PM (BC-PIV/S-2PM)	This paper	N/A
pCAGGS-PIV2 NP	Ohtsuka et al. (2014)	N/A
pCAGGS-PIV2 P	Ohtsuka et al. (2014)	N/A
pCAGGS-PIV2 L	Ohtsuka et al. (2014)	N/A
SRα-T7	Ohtsuka et al. (2014)	N/A

**Software and algorithms**		

FlowJo (version 7.2.5)	BD Biosciences	N/A
R (version 4.0.2)	The R Foundation	https://www.r-project.org/

### Resource availability

#### Lead contact

Further information and requests for resources and reagents should be directed to and will be fulfilled by the lead contact, Tetsuya Nosaka (nosaka@doc.medic.mie-u.ac.jp).

#### Materials availability

Requests for material can be directed to Masayuki Fukumura (m-fukumura@biocomo.jp) and the lead contact, Tetsuya Nosaka (nosaka@doc.medic.mie-u.ac.jp). All materials and reagents will be made available upon installment of a material transfer agreement (MTA).

### Experimental model and subject details

#### Cell lines and cell culture

Vero cell line was obtained from RIKEN BioResource center (Tsukuba, Japan), and cultured in a minimal essential medium (Sigma) supplemented with 10% heat-inactivated fetal bovine serum (FBS) (GIBCO/Invitrogen). Vero/BC-F cells (Ohtsuka et al., 2014) were established by our group, and used for proliferation of BC-PIV vectors as described later. VeroE6/TMPRSS2 cells (Matsuyama et al., 2020) (JCRB 1819) were propagated in the presence of 1 mg/ml Geneticin (G418; InvivoGen) and 5 μg/ml Plasmocin prophylactic (InvivoGen) in Dulbecco’s Modified Eagle’s Medium (DMEM) containing 10% FBS and antibiotics.

#### Animals

Female, 5-6-week-old BALB/c mice were purchased from CLEA Japan Inc., and female, 4-weel-old Syrian hamsters were purchased from Japan SLC. All animals were housed individually under specific pathogen-free conditions in a temperature control environment with a 12 h: 12h light: dark cycle, with 75–80% humidity and ad libitum access to water and standard laboratory chow. All animal studies were approved by the Animal Care Committees of Mie University (Approved No. 23-33) and the Animal Experiment Committee of the Institute of Medical Science, the University of Tokyo (Approved No. PA19-75), and all methods were performed under institutional regulations of animal experiments in accordance with the current national guidelines. Animal experiments using SARS-CoV-2 *S* gene fragments or SARS-CoV-2 were also approved by the Ministry of Education, Culture, Sports, Science and Technology in Japan (Approved No. 2019-728, 729; 2020-362, 373, 2020-948).

#### Viruses

NC002, MD-HP0154, and UW-5250 were propagated in VeroE6/TMPRSS2 cells in VP-SFM (Thermo Fisher Scientific). UT-NCGM02 (Imai et al., 2020) was propagated in VeroE6 cells in Opti-MEM I (Thermo Fisher Scientific) containing 0.3% bovine serum albumin and 1 μg of L-1-Tosylamide-2-phenylethyl chloromethyl ketone-trypsin/ml. All experiments with SARS-CoV-2 were performed in enhanced biosafety level 3 containment laboratories at the University of Tokyo, which are approved for such use by the Ministry of Agriculture, Forestry, and Fisheries, Japan.

### Method details

#### Fusion induction assay

The wild-type *S* gene of SARS-CoV-2 or its 2P mutant subcloned in pcDNA3.1 was transfected into Vero cells using an X-treme GENE HP (Roche). Forty-eight hours post-transfection, cells were fixed with 4% paraformaldehyde (PFA) in phosphate-buffered saline (PBS) (Nacalai Tesque) and permeabilized with 0.1% Triton-X (Wako Pure Chemical Industries, Ltd.)/PBS. The effects of the S protein to induce syncytia were visualized using a BZ-X810 all-in-one fluorescence microscope (Keyence) after immunohistochemical staining. A rabbit anti-SRAS-CoV S1 antibody (Sino Biological Inc.) and Alexa 488-conjugated goat anti-rabbit IgG antibody (Abcam) were used as primary and secondary antibodies, respectively, with 4′,6-diamidino-2-phenylindole (DAPI) (Nacalai Tesque) staining.

#### Construction of the plasmids

An entire fragment of SARS-CoV-2 (Wuhan/WIV04/2019) *S* (*S*) gene (Sino Biological Inc.) with optimized codon usage (the translated amino acid sequence is identical to GenBank: QHD43416.1) was inserted into the pcDNA3.1+ (Invitrogen/Thermo Fisher Scientific). Prefusion-stabilized 2P mutations (K986P/V987P) were introduced into the *S* gene with conventional polymerase chain reaction (PCR)-based methods.

For recombinant BC-PIV vaccines, four kinds of *S* fragments were produced: full-length *S* with 2P mutations, named S-2P; a chimeric version of S-2P created by the substitution of the transmembrane (TM) and cytoplasmic tail (CT) regions with those of the hPIV2 *F* gene (IALILSVITLVVVGLLIAYIIKLVSQIHQFRSLAATTMFHRENPAFFSKNNHGNIYGIS), named S-2PM; S1 domain alone, named S1; and S1 domain followed by hPIV2 F TMCT regions, named S1M. Each fragment of these four versions was sequenced and cloned into the cloning site 1 (NotI) of the BC-PIV plasmid (Ohtsuka et al., 2019), which is just 5’ to the most upstream gene *NP* in the genome. The insert fragment consists of the sequences as follows; 5’-NotI-A/ATAT (for S1M, S-2PM/for S1, S2-P; these nucleotides were used to adjust to the rule of six (Kolakofsky et al., 1998), respectively)-ATG・・・TAA-CTCTCATAATTTAAGAAAAAATCATAGGCCCGGACGGGTTAG-NotI-3’. BC-PIV is derived from Toshiba strain of hPIV2 (GenBank accession number AB176531). Recombinant DNA experiments with SARS-CoV-2 *S* gene fragments were approved by the Ministry of Education, Culture, Sports, Science and Technology in Japan (Approved No. 2019-728, 729; 2020-362, 373, 948). The sequences and detailed construction procedures of the plasmids are available upon request.

#### Virus production from the vaccine vector

The production of a virus seed and its propagation have been described previously (Ohtsuka et al., 2014). In brief, the packaging cell line Vero/BC-F which stably expresses F protein of hPIV2 on 6-well plates was transfected with the recombinant phPIV2ΔF (= plasmid for BC-PIV) (10 μg), along with each expression plasmid encoding the hPIV2 *NP* (2 μg), P (0.9 μg), *L* (2 μg), and *T7* RNA polymerase (3 μg), using an X-treme GENE HP (Roche) according to the manufacturer’s instructions. One week later, the supernatant was centrifuged to remove cells and cell debris and transferred to fresh Vero/BC-F cell culture in minimal essential medium with 1% heat-inactivated FBS for virus propagation. The virus solutions were concentrated by ultracentrifugation (141,000 *g*, 4°C, 30 min) and dissolved in PBS. Subsequently, the virus titer was determined by the median tissue culture infective dose (TCID_50_) method using Vero/BC-F cells on 96-well plates.

#### Western blotting of the BC-PIVs and immunohistochemical analyses of S1 protein of the BC-PIV vector-infected cells

Vero/BC-F cells were infected with the recombinant BC-PIV vectors. The viruses recovered by ultracentrifugation (141,000 *g*, 4°C, 30 min) of the supernatants and cells were harvested 7 days post-infection. The cells and viral particles were each dissolved in the reducing sample buffer (Nacalai Tesque), heated for 5 min at 100°C, and separated on a 5-20% gradient sodium dodecyl sulfate-polyacrylamide gel electrophoresis (SDS-PAGE) gel (FUJIFILM Wako Pure Chemical). Protein was transferred to a nitrocellulose membrane and incubated for 1 h at room temperature (RT) in 10% (W/V) skim milk in PBS. The membrane was then incubated at 4°C overnight in PBS containing a rabbit polyclonal anti-SARS-CoV S1 antibody that cross-reacts to SARS-CoV-2 S1 (1:1,000 dilution; Sino Biological Inc.) or a mouse monoclonal anti-SARS-CoV S2 antibody (1A9) that cross-reacts to SARS-CoV-2 S2 (1:1,000 dilution; GeneTex). The membrane was washed three times with PBS containing 0.05% Tween20 (Wako Pure Chemical Industries, Ltd.) (PBST) and incubated for 1 h at RT with a horseradish peroxidase (HRP)-conjugated goat anti-rabbit IgG or horse anti-mouse IgG antibody (1:1,000 dilution; Cell Signaling Technology) in PBS. Finally, the membrane was washed three times with PBST, and the protein was visualized using a Western Blotting Luminol Reagent (Santa Cruz Biotechnology). After detection of SARS-CoV-2 S proteins, membranes were re-probed with a mouse anti-hPIV2 NP monoclonal antibody (20A) or a mouse anti-hPIV2 P monoclonal antibody (211A) (Tsurudome et al., 1989) and then incubated with HRP-conjugated anti-mouse IgG. An HRP-conjugated mouse anti-GAPDH (glyceraldehyde-3-phosphate dehydrogenase) monoclonal antibody (FUJIFILM Wako Pure Chemical) was used as a loading control in cell lysates.

To detect secreted S1 protein, Vero cells were infected with BC-PIV/S1 or BC-PIV at MOI of 3. Three days after infection, culture supernatant was centrifuged at 1,000 g for 5 min to remove cell debris, and the supernatant was filtrated through a 0.22 μm pore-size membrane filter (Merck Millipore). After ultracentrifugation of the filtrate at 141,000 g for 30 min at 4°C, the pellet was dissolved in the reducing sample buffer. The supernatant after the ultracentrifugation was mixed with the equal volume of acetone (Wako Pure Chemical Industries, Ltd.), put overnight at -20°C, and centrifuged at 13,000 g for 10 min at 4°C. The pellet was dissolved in the reducing sample buffer, and was used as “supernatant”. The samples from each fraction were subjected to western blot analysis. SARS-CoV-2 S1 protein and hPIV2 P and V proteins that have common N-terminal structure with different C-terminal structure due to RNA editing, were detected with an anti-SARS-CoV-2 S1 antibody (1:1,000 dilution, Sino Biological Inc.) and an anti-hPIV2 P/V antibody (315-1, 1:500 dilution) (Nishio et al., 1997), respectively.

To detect the SARS-CoV-2 S protein by immunohistochemical analyses, BC-PIV-based vaccine-infected Vero cells or SARS-CoV-2 S expression vector-transfected Vero cells were fixed with 4% PFA in PBS at RT for 15 min and permeabilized with 0.1% Triton-X/PBS at RT for another 15 min. SARS-CoV-2 S and hPIV2 NP proteins were detected using the rabbit anti-SARS-CoV S1 antibody and the mouse anti-hPIV2 NP antibody (20A), respectively, followed by an Alexa 568-conjugated anti-rabbit IgG antibody and an Alexa 488-conjugated anti-mouse IgG antibody (Abcam), respectively. Nuclei were visualized with DAPI staining (Nacalai Tesque). Immuno-stained cells were observed under a BZ-X810 all-in-one fluorescence microscope.

#### Electron microscopy

Vero/BC-F cells on coverslips were infected with BC-PIV/S-2PM or BC-PIV at a multiplicity of infection (MOI) of 0.1. Three days after infection, cells were fixed with a mixture of 4% PFA and 2.5% glutaraldehyde (GA) (Nacalai Tesque) in PBS at RT for 1 h. After washing with 0.1 M phosphate buffer (PB), the cells were post-fixed with 1% OsO_4_ for 1 h at RT, dehydrated with graded alcohol, and embedded in epoxy resin. Ultrathin sections (50 nm) were cut, mounted on a grid, and contrasted with 2% uranyl acetate in 50% alcohol and lead citrate. Electron micrographs were taken at 100 kV on a transmission electron microscope (JEM-1400Plus; JEOL, Tokyo, Japan).

#### Immunoelectron microscopy

BC-PIV/S-2PM or BC-PIV was infected to Vero/BC-F cells on cover slips at MOI of 0.1. Three days after infection, cells were fixed with a mixture of 2% PFA and 0.1% GA in PBS at RT for 30 min and post-fixed with 4% PFA in PB for overnight at 4°C. After washing with PB, the cells were incubated with PBS containing 10% normal goat serum for 1 h at RT for blocking and incubated overnight at 4°C with the anti-SARS-CoV-2 S1 antibody (1:300 dilution, Sino Biological Inc.). After washing with PB, the cells were incubated with a biotinylated anti-rabbit IgG (1:200; Vector Laboratories) for 3 h at RT. After washing with PB, the cells were incubated with VECTASTAIN ABC reagent (Vector Laboratories) for 3 h at RT and visualized by 3,3’-diaminobenzidine tetrahydrochloride (DAB)-H_2_O_2_ solution. After the DAB reaction, cells were post-fixed with 1% OsO4 for 1 h at RT, dehydrated and embedded to prepare an Epon block. Serial ultrathin sections (50 nm) were cut, mounted on a grid, and contrasted with 2% uranyl acetate and lead citrate. Immunoreaction was identified and imaged with a transmission electron microscope (JEM-1400Plus; JEOL).

#### Binding assay between the S protein on the recombinant BC-PIV vectors and human ACE2

Recombinant BC-PIVs (1×10^6^ TCID_50_) in PBS on the 96-well plates were immobilized at 4°C overnight. After immobilization, the plates were incubated with 4% Block Ace (DS Pharma Biomedical)/PBS for 2 h at 4°C. After blocking, serially diluted recombinant human ACE2 protein with Fc Tag (ACROBiosystems) was added to each well, and the plate was incubated for 2 h at 4°C, followed by washing 6 times with PBST and subsequent 1-h incubation with HRP-conjugated protein A (1:1,500 dilution; BioLegend) in PBS in the dark at RT. The plates were then washed further 6 times with PBST, and 100 μL of TMB Start solution of ELISA POD substrate TMB solution (Nacalai Tesque) was added to each well. The plate development was halted by the addition of 50 μL of 1N H_2_SO_4_ per well. The absorbance at 450 nm was recorded using a microplate reader.

#### Hemagglutination assay induced by hPIV2

The viral suspension was twofold serially diluted with cold PBS on ice. The diluted viral suspension was mixed with equal volume of 2% guinea pig erythrocytes in a 96-well U-bottom plate and incubated at 4°C for 1 h. The hemagglutination units were then evaluated.

#### Mouse study

For mouse experiments, recombinant BC-PIVs or a control empty vector (2×10^7^ TCID_50_/20 μl of PBS) was administered to 6- to 7-week-old female BALB/c mice (CLEA Japan Inc.) via an intranasal (IN) route using a pipette tip. A subset of the immunized mice were boosted under the same conditions four weeks after the first shot. Thirty-three or 35 days after the first shot, all mice were sacrificed, and sera and nasal washes were collected for analyses.

#### Enzyme-linked immunosorbent assays for mouse sera and nasal washes

Ninety-six-well plates were coated with 1 μg/ml SARS-CoV-2 S1 protein (Sino Biological Inc.) or S1 RBD protein (Sino Biological Inc.) in PBS and incubated at 4°C overnight. After coating, plates were treated with 2% bovine serum albumin (BSA) (Sigma)/PBS for 2-5 h at 4°C. After blocking, serially diluted heat-inactivated sera or nasal washes (1%BSA/PBS) from the vaccinated or control mice were added to wells, and the plates were incubated for 1.5 h at RT, followed by washing 6 times with PBST and subsequent 1h incubation with an HRP-conjugated goat anti-mouse IgG (1:3,000 dilution; BioLegend) or goat anti-mouse IgA antibody (1:5,000 dilution; SouthernBiotech) in 1% BSA/PBS at RT. The wells were washed 6 times with PBST, and 100 μL of the TMB substrate set (BioLegend) was added to the immune complexes in each well. Plate development was then halted by the addition of 50 μL of 2N H_2_SO_4_ per well. The absorbance at 450 nm was recorded using a microplate reader. The ELISA endpoint titers were defined as the highest reciprocal serum dilution that yielded an absorbance >0.2. (Log_10_ endpoint titers were given).

#### SARS-CoV-2 surrogate virus neutralization test

A surrogate test for SARS-CoV-2 neutralization based on the antibody-mediated blockage of the interaction of ACE2 and receptor-binding domain of the S protein (Tan et al., 2020), was performed using a SARS-CoV-2 surrogate virus neutralization test kit (GenScript). In brief, serially diluted vaccinated or control mice sera were incubated with HRP-conjugated SARS-CoV-2 S RBD protein at 37°C for 30 min, and the mixture was incubated with the human ACE2 protein immobilized on the plate at 37°C for 15 min. TMB solution was then added to the mixture in each well, and the reaction was halted by adding the stop solution. The absorbance at 450 nm was recorded using a microplate reader. The percent inhibition was calculated as follows: [1 – (OD of the sample / OD of the negative control)] × 100(%).

#### Intracellular staining of IFN-γ in splenocytes of the vaccinated mice after stimulation with SARS-CoV-2 S protein peptide library

Splenocytes (3.0×10^6^ cells) from the vaccinated or control mice were stimulated with the SARS-CoV-2 S protein peptide library (GenScript) (1 μg/mL, 15-mer peptides overlapping with 11 amino acids, covering the entire S protein of SARS-CoV-2) and anti-mouse CD28 antibody (1 μg/mL, BioLegend) in the presence of Brefeldin A (1 μg/mL, BD Biosciences) for 6 h at 37°C.

Intracellular staining was performed using the eBioscience™ Intracellular Fixation & Permeabilization Buffer Set (Thermo Fisher Scientific) according to the manufacturer’s protocol. In brief, the stimulated cells were stained with peridinin chlorophyll protein (PerCP)-Cy5.5-conjugated anti-CD4 (GK1.5) or anti-CD8a (53-6.7) monoclonal antibodies, fixed with IC fixation buffer, permeabilized with 1x Permeabilization Buffer and then stained with phycoerythrin (PE)-conjugated anti-IFN-γ monoclonal (XMG1.2) antibody. A FACS analysis was performed using a FACS Canto II (BD Biosciences). Data were analyzed with the FlowJo software program, version 7.2.5 (BD Biosciences).

#### SARS-CoV-2 challenge test in BC-PIV-vaccinated hamsters

Five-week-old female Syrian hamsters (Japan SLC) were intra-nasally vaccinated with BC-PIV/S-2PM or an empty vector BC-PIV (1.0×10^8^ TCID_50_/100 μl of PBS). Nine weeks after the vaccination, half of the BC-PIV/S-2PM-treated hamsters were subjected to the booster vaccination with the same dose as a prime shot. Eleven weeks after the prime shot, all hamsters were intra-nasally infected with 10^3^ plaque-forming units (PFU) of SARS-CoV-2 (UT-NCGM02/Human/2020/Tokyo)/30 μl of PBS. At day 3 post-infection, the hamsters were sacrificed to analyze the virus titers in the lungs and nasal turbinates by plaque assays on VeroE6/TMPRSS2 cells constitutively expressing transmembrane protease serine 2 (TMPRSS2), which activates SARS-CoV-2 infection (Matsuyama et al., 2020).

#### Pathological examinations

Excised lungs of the hamsters were fixed in 4% PFA/PB, and processed for paraffin embedding. The paraffin blocks were cut into 3 μm-thick sections and then mounted on silane-coated glass slides. One section from each tissue sample was stained using a standard hematoxylin and eosin procedure. Another was processed for immunohistochemical staining with a rabbit polyclonal antibody for SARS-CoV-1 nucleocapsid protein (Prospec; ANT-180), which cross-reacts with SARS-CoV-2 nucleocapsid protein. Specific immune reactions were visualized by DAB staining using the Envision detection system (Dako).

#### Authentic SARS-CoV-2 neutralization assay

Sera of the vaccinated mice were incubated at 56°C for 30 min. Thirty-five μL of the virus (140 tissue culture infectious dose 50) was incubated with 35 μL of twofold serially diluted serum for 1 h at RT, and 50 μL of the mixture was added to confluent VeroE6/TMPRSS2 cells in 96-well plates, and incubated for 1 h at 37°C. After the addition of 50 μL of DMEM containing 5% FBS, the cells were incubated for 3 more days at 37°C. Viral cytopathic effects (CPE) were observed under an inverted microscope, and virus neutralization titers were determined as the reciprocal of the highest serum dilution that completely prevented the CPE.

### Quantification and statistical analysis

Statistical analyses of the results of the ELISAs (Figures 5C, 5D, and 6B–6D) were performed on the values after logarithmic conversion. First the assumption of homogeneity of variance among the 4-6 groups was tested using the Kruskal-Wallis rank sum test, and the p-values of the results of Figures 5C, 5D, 6B-S1, 6B-RBD, 6C-S1, 6C-RBD, 6D-S1, and 6D-RBD were 0.00004319, 0.0002989, 0.03143, 0.03341, 0.0002402, 0.0002707, 0.0002928, and 0.00003737, respectively. Multiple comparisons between two groups were then performed using the Dunn’s post-hoc test with Holm-Bonferroni p-value adjustment.

Statistical analyses of the results of the plaque-forming assay (Figure 7C) were performed on the values after logarithmic conversion. Values that were not detected (ND) were not plotted in the figure. However, those in nasal turbinates (n = 2, ND, ND) were set as 1.0 in statistical analyses along with the other two values (n = 2, 1.6, 1.5). First the assumption of homogeneity of variance among the three groups was tested using the Kruskal-Wallis rank sum test, and the p-value was 0.007029. A comparison between two groups was then performed using the Steel-Dwass test as a post-hoc multiple comparison test. All statistical tests were performed using the R software program, version 4.0.2 (https://www.r-project.org/).

#### Additional information

Supplemental information is available for this paper.

## Data Availability

This study did not generate any unique datasets or code. All data reported in this paper will be shared by the lead contact upon request. Raw data of all figures in this paper are available at: https://data.mendeley.com/datasets/dhzdv7by5y/1.

## Footnotes

Supplemental information can be found online at https://doi.org/10.1016/j.isci.2021.103379.
