# Supplementary material for: Non-propagative human parainfluenza virus type 2 nasal vaccine robustly protects the upper and lower airways against SARS-CoV-2
Source: iScience. 2021 Nov 17;24(12):103379. doi: 10.1016/j.isci.2021.103379 (PMC8596570; doi:10.1016/j.isci.2021.103379)
Supplement: Document S1. Figures S1 and S2 [file mmc1.pdf]

iScience, Volume *24*

Supplemental information

Non-propagative human parainfluenza virus type 2 nasal vaccine robustly protects the upper and lower airways against SARS-CoV-2

Junpei Ohtsuka, Masaki Imai, Masayuki Fukumura, Mitsuyo Maeda, Asami Eguchi, Ryoichi Ono, Tadashi Maemura, Mutsumi Ito, Seiya Yamayoshi, Yosky Kataoka, Yoshihiro Kawaoka, and Tetsuya Nosaka

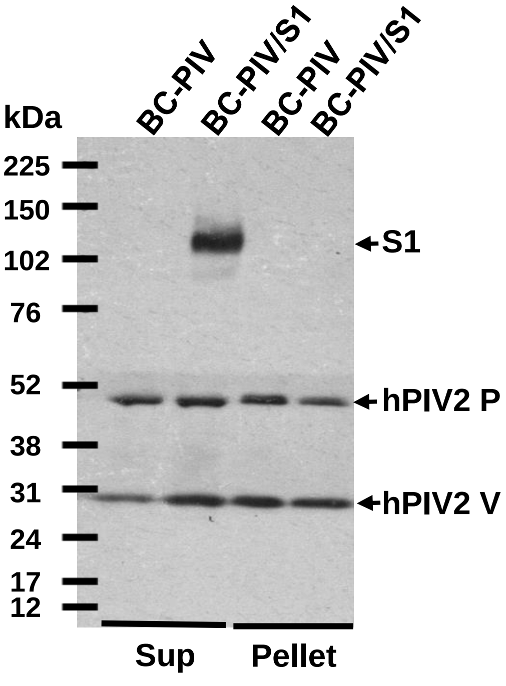

Figure S1. Secretion of S1 protein into the culture supernatant after infection of BC-PIV/S1 to Vero cells, related to Figure 2. The culture supernatant was used to detect secreted S1 protein. Since the culture supernatant contains small amount of the viral proteins derived from the disrupted cells, the supernatant fraction as well as pellet fraction containing viral particles shows the bands of the hPIV2 P/V proteins. S1 protein is detected only in the supernatant fraction.

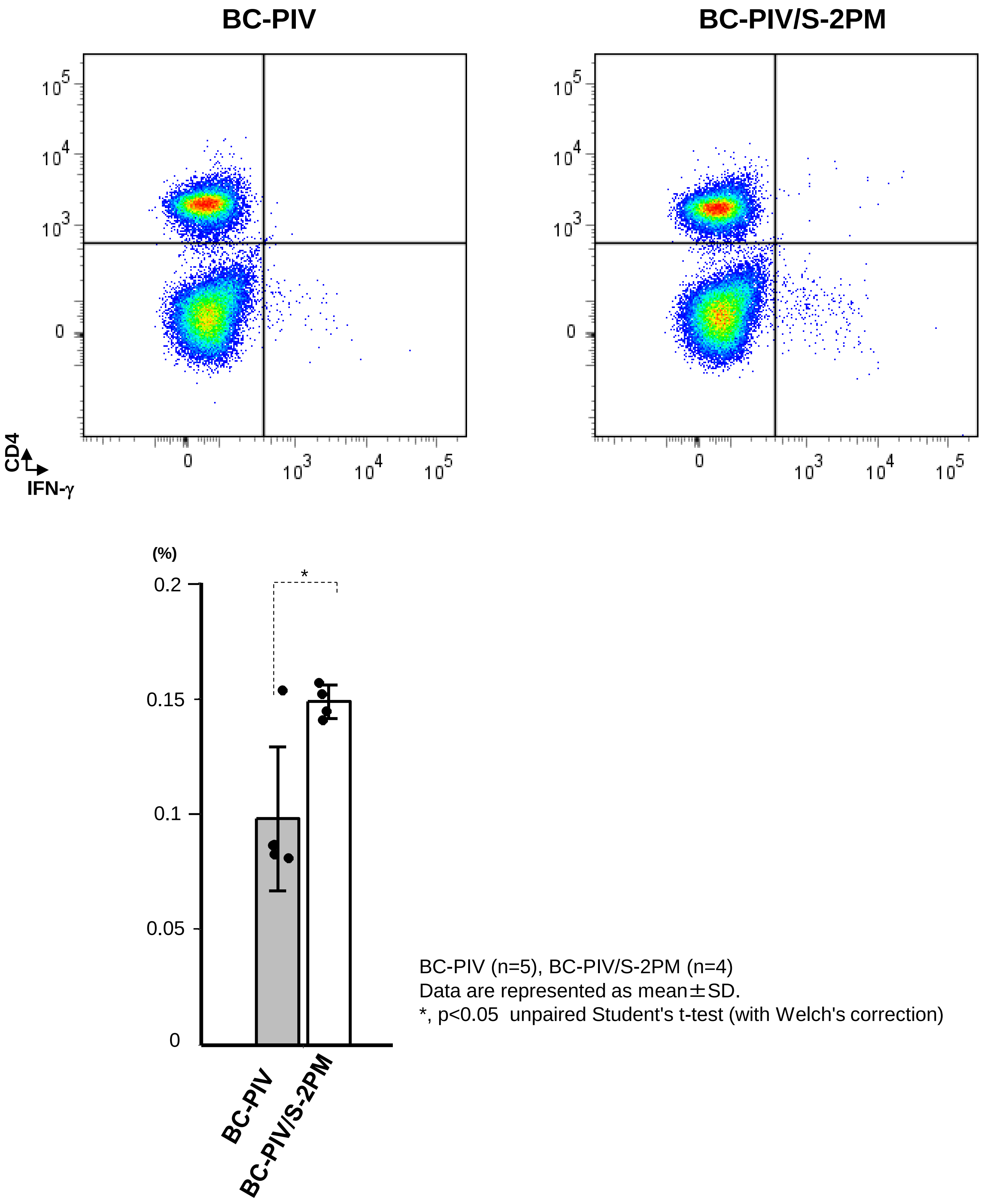

Figure S2. Modest induction of intracellular IFN-γ in CD4+ splenocytes from intranasally vaccinated mice with BC-PIV/S-2PM after stimulation with the SARS-CoV-2 S peptide library *in vitro*, related to STAR Methods. Three independent experiments with prime and boost immunization gave similar results, and one representative result is shown. The timeline and protocol are the same as those shown in Figure 6A.
